# Magnetic moment of inertia within the torque-torque correlation model

**DOI:** 10.1038/s41598-017-01081-z

**Published:** 2017-04-19

**Authors:** Danny Thonig, Olle Eriksson, Manuel Pereiro

**Affiliations:** Department of Physics and Astronomy, Material Theory, University Uppsala, S-75120 Uppsala, Sweden

## Abstract

An essential property of magnetic devices is the relaxation rate in magnetic switching which strongly depends on the energy dissipation. This is described by the Landau-Lifshitz-Gilbert equation and the well known damping parameter, which has been shown to be reproduced from quantum mechanical calculations. Recently the importance of inertia phenomena have been discussed for magnetisation dynamics. This magnetic counterpart to the well-known inertia of Newtonian mechanics, represents a research field that so far has received only limited attention. We present and elaborate here on a theoretical model for calculating the magnetic moment of inertia based on the torque-torque correlation model. Particularly, the method has been applied to bulk itinerant magnets and we show that numerical values are comparable with recent experimental measurements. The theoretical analysis shows that even though the moment of inertia and damping are produced by the spin-orbit coupling, and the expression for them have common features, they are caused by very different electronic structure mechanisms. We propose ways to utilise this in order to tune the inertia experimentally, and to find materials with significant inertia dynamics.

## Introduction

The research on magnetic materials with particular focus on spintronics or magnonic applications became more and more intensified, over the last decades^1, 2^. For this purpose, “good” candidates are materials exhibiting thermally stable magnetic properties^3^, energy efficient magnetisation dynamics^4, 5^, as well as fast and stable magnetic switching^6, 7^. Various magnetic excitation methods^8–10^ allow switching of the magnetic moment on sub-ps timescales.

**Figure 1:**
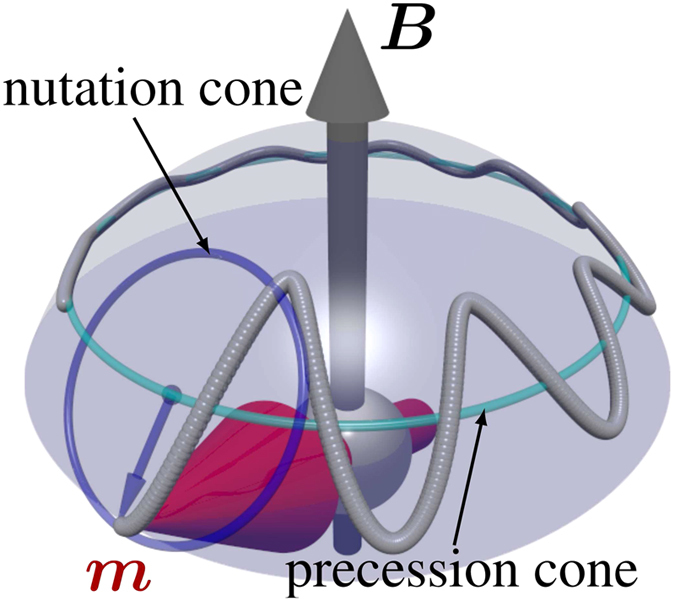
Schematic figure of the atomistic magnetic moment evolution effected by magnetic inertia. Without inertia and damping, the out-of-equilibrium magnetic moment ***m*** (red arrow) precesses around an effective magnetic field ***B*** (grey arrow), represented by a bright blue trajectory. Magnetic inertia, however, results in a precession around the angular momentum axis (dark blue line), that moves along the bright blue line. The resulting, superimposed trajectory (grey line) of the magnetic moment shows nutation. Since damping and inertia exists on different time scales, only the damping of the nutation but not the damping in effective magnetic field direction is shown.

On the theoretical side it has been argued that the classical atomistic Landau-Lifshitz-Gilbert (LLG) equation^11, 12^ should be relevant over a time-scale of sub-pico seconds and longer^13^ and provides a proper description of magnetic moment switching^14^, but is derived within the adiabatic limit^15, 16^. This limit characterises the blurry boundary where the time scales of electrons and atomic magnetic moments are separable^17^ — usually between 10–100 fs. In this time-scale, the applicability of the atomistic LLG equation must be scrutinised in great detail. On one hand, the quantum mechanical equation of motion can be solved^18, 19^, but on the other hand one can linger with the classical approach and instead introduce higher order terms in the LLG. In its common formulation, the LLG equation does not account for longitudinal fluctuations of magnetic moment^20^, quantum mechanical spin currents^21^ or, in particular, the influence of magnetic inertia^22^, compared to its classical mechanical counterpart of a gyroscope. If the rotation axis of the gyroscope do not coincide with the angular momentum axis due to a “fast” external force, a superimposed precession around the angular-momentum and the gravity field axis occurs; the gyroscope nutates. In coexistence with damping, this nutation disappears on a short time. It may be expected that for magnetisation dynamics that atomic magnetic moments behave in an analogous way on ultrafast time-scales^22, 23^ (Fig. 1). In this work, we provide a theoretical foundation for understanding inertia in magnetisation dynamics, based on electronic structure theory. We obtain numerical results that are consistent with experimental values, and elaborate on how inertia can be detected in magnetic materials.

Conceptional thoughts related to the velocity of moving a static domain wall with “inertial mass” were already introduced by Döring^24^, De Leeuw and Robertson^25^. More recently, nutation was discovered on a single-atom magnetic moment trajectory in a Josephson junction^26–28^. From micromagnetic Boltzman theory, Ciornei *et al*.^23^ derived a term in the extended LLG equation that addresses “inertial magnetic mass” by including the moment of inertia tensor *ι* into the equation of motion. This macroscopic model was further analysed numerically in ref. 22, and analytically in refs 29 and 30. Like the Gilbert damping *α*, the moment of inertia tensor *ι* has been considered as a material specific parameter in theoretical investigations. Recently, the moment of inertia was experimentally examined by Li *et al*.^31^ who measured it for Ni_79_Fe_21_ and Co films near room temperature with ferromagnetic resonance (FMR) in the high-frequency regime (around 200 GHz). At these high frequencies, an additional stiffening of the FMR resonance was observed that was quadratic in the probing frequency *ω* and, consequently, proportional to the moment of inertia. In these experiments, the finite electron (Bloch-state) lifetime *τ* was determined to be in the range of *τ* = 0.12–0.47 ps, depending not only on the selected material but also on its thickness. This result, and more generally, experimental investigations of magnetisation dynamics that focus on inertia effects, calls for a proper theoretical description and calculations based on *ab*-*initio* electronic structure footings.

A first model was already provided by Bhattacharjee *et al*.^32^, where the moment of inertia *ι* was derived in terms of Green’s functions in the framework of the linear response theory. However, neither first-principles electronic structure-based numerical values nor a detailed physical picture of the origin of the inertia and a potential coupling to the electronic structure was reported in this study. In this article, we derive a model for the moment of inertia tensor based on the torque-torque correlation formalism^33, 34^. We reveal the basic electron mechanisms for observing magnetic inertia by calculating numerical values for bulk itinerant magnets Fe, Co, and Ni with both the torque-torque correlation model and the linear response Green’s function model^32^. Interestingly, our study elucidate also the misconception about the sign convention of the moment of inertia^35^.

The moment of inertia *ι* is defined in a similar way as the Gilbert damping *α* within the effective dissipation field *B*_diss_^33, 36^. This *ad hoc* introduced field is expanded in terms of viscous damping ${\alpha }^{\partial {\boldsymbol{m}}}/\partial t$ and magnetic inertia ${\iota }^{{\partial }^{2}{\boldsymbol{m}}}/\partial {t}^{2}$ in the relaxation time approach^35, 37^ (see Supplementary Material), where ***m*** is the magnetic moment. The off-equilibrium magnetic state induces excited states in the electronic structure due to spin-orbit coupling. Within the adiabatic limit, the electrons equilibrate into the ground state at certain time scales due to band transitions^38^. If this relaxation time *τ* is close to the adiabatic limit, it will have two implications for magnetism: *i*) magnetic moments respond in a inertial fashion, *ii*) the kinetic energy is proportional to ${\rm{m}}{{\boldsymbol{u}}}^{2}/2$ with the velocity ${\boldsymbol{u}}=\partial {\boldsymbol{m}}/\partial t$ and the “inertial mass” of magnetic moments, following equations of motion of classical Newtonian mechanics. The inertia forces the magnetic moment to remain in their present state, represented in the Kamberský model by a relationship between inertia, damping and relaxation time, as *ι* = −*α* · *τ* (refs 35 and 37 and confirmed here by us (see Supplementary Material)).

In experiments, the Gilbert damping and the moment of inertia can be shown to be measurable from the diagonal elements of the magnetic response function *χ* via ferromagnetic resonance^34^ (see Supplementary Material). We derive that, in particular for the moment of inertia,

1 $$\alpha =\frac{{\omega }_{0}^{2}}{{\omega }_{M}}\mathop{\mathrm{lim}}\limits_{\omega \to 0}\frac{\Im {{\chi }}^{\perp }}{\omega }$$

2 $$\iota =\frac{1}{2}\frac{{\omega }_{0}^{2}}{{\omega }_{M}}\mathop{\mathrm{lim}}\limits_{\omega \to 0}\frac{{\partial }_{\omega }\Re {\chi }^{\perp }}{\omega }-\frac{1}{{\omega }_{0}},$$

where *ω*_*M*_ = *γB* and *ω*_0_ = *γB*_0_ are the frequencies related to the internal effective (*B*) and the external magnetic field (*B*_0_), respectively. Thus, the moment of inertia *ι* is equal to the change of the FMR peak position, say the first derivative of the real part of *χ*, with respect to the probing frequency^32, 39^. Alternatively, rapid external field changes induced by spin-polarised currents lead also to nutation of the macrospin.

Setting *χ* on *ab*-*initio* footings, the Kubo-Greenwood formula has to be evaluated^40, 41^. We do this by using the torque-torque correlation model, as applied for the Gilbert damping in refs 33 and 38. Here, we derive a one-to-one mapping between the first derivative of the Kubo-Greenwood susceptibility and of the frequency dependent Green’s function of the correlation between the torques *T* ^−^ and *T* ^+^, i.e. ${\mathscr{G}}(\omega )=-{\rm{i}}/\hslash \int {\rm{d}}\tau {{\rm{e}}}^{{\rm{i}}\omega \tau }{\rm{\Theta }}(\tau )\langle \langle {T}^{-}(\tau ),{T}^{+}(\tau )\rangle \rangle$. It is shown (see Supplementary Material) that the time dependence of the torque operator is handled by a unitary transformation and, thus, the matrix elements of the torque operator ${T}_{nm}^{\pm }$ can be separated from the energy integration. The derivative with respect to *ω* acts only on the spectral overlap between the bands ${U}_{n,m}({\boldsymbol{k}},\omega )=\int {\rm{d}}{\varepsilon }_{1}\int {\rm{d}}{{\varepsilon }_{2}}^{f({\varepsilon }_{1})-f({\varepsilon }_{2})}/(\hslash \omega +{\varepsilon }_{1}-{\varepsilon }_{2}+{\rm{i}}\delta ){A}_{n{\boldsymbol{k}}}({\varepsilon }_{1}){A}_{m{\boldsymbol{k}}}({\varepsilon }_{2})$. Finally, we obtain (see Supplementary Material)

3 $${\alpha }^{\mu \nu }=\frac{g\pi }{{m}_{s}}\sum _{nm}\int {T}_{nm}^{\mu }({\boldsymbol{k}}){T}_{nm}^{\ast \nu }({\boldsymbol{k}}){W}_{nm}{\rm{d}}{\boldsymbol{k}}$$

4 $${\iota }^{\mu \nu }=-\frac{g\hslash }{{m}_{s}}\sum _{nm}\int {T}_{nm}^{\mu }({\boldsymbol{k}}){T}_{nm}^{\ast \nu }({\boldsymbol{k}}){V}_{nm}{\rm{d}}{\boldsymbol{k}},$$

where *μ*, *ν* = *x*, *y*, *z* and *m*_*s*_ is the size of the magnetic moment. The spin-orbit-torque matrix elements ${{\boldsymbol{T}}}_{nm}=\langle n,{\boldsymbol{k}}|{\boldsymbol{[}}{\boldsymbol{\sigma }},{H}_{soc}]|m,{\boldsymbol{k}}\rangle$ — related to the commutator of the Pauli matrices ***σ*** and the spin-orbit Hamiltonian — create transitions between electron states |*n*, ***k***〉 and |*m*, ***k***〉 in bands *n* and *m*. It is remarkable that this mechanism is equal for both the Gilbert damping and moment of inertia. Note that the wave vector ***k*** is conserved, since we neglect non-uniform magnon creation with non-zero wave vector. The difference between moment of inertia and damping comes from different weighting mechanism *W*_*nm*_, *V*_*nm*_: for the damping ${W}_{nm}=\int \eta (\varepsilon ){A}_{n{\boldsymbol{k}}}(\varepsilon ){A}_{m{\boldsymbol{k}}}(\varepsilon ){\rm{d}}\varepsilon$ where the electron spectral functions are represented by Lorentzian’s *A*_*n****k***_(*ε*) centred around the band energies *ε*_*n****k***_ and broadened by interactions with the lattice, electron-electron interactions or alloying. The width of the spectral function ${\rm{\Gamma }}$ provides a phenomenological account for angular momentum transfer to other reservoirs. For inertia, however, ${V}_{nm}=\int f(\varepsilon )({A}_{n{\boldsymbol{k}}}(\varepsilon ){B}_{m{\boldsymbol{k}}}(\varepsilon )+{B}_{n{\boldsymbol{k}}}(\varepsilon ){A}_{m{\boldsymbol{k}}}(\varepsilon )){\rm{d}}\varepsilon$ where ${B}_{m{\boldsymbol{k}}}(\varepsilon )=\mathrm{2(}\varepsilon -{\varepsilon }_{m{\boldsymbol{k}}})({(\varepsilon -{\varepsilon }_{m{\boldsymbol{k}}})}^{2}-3{{\rm{\Gamma }}}^{2})/{({(\varepsilon -{\varepsilon }_{m{\boldsymbol{k}}})}^{2}+{{\rm{\Gamma }}}^{2})}^{3}$ (see Supplementary Material). Here, *f*(*ε*) and $\eta (\varepsilon )$ are the Fermi-Dirac distribution and the first derivative of it with respect to *ε*. Knowing the explicit form of *B*_*m****k***_, we can reveal particular properties of the moment of inertia: *i*) for ${\rm{\Gamma }}\to 0$ ($\tau \to \infty$), ${V}_{nm}=2/{({\varepsilon }_{n{\boldsymbol{k}}}-{\varepsilon }_{m{\boldsymbol{k}}})}^{3}$. Since *n* = *m* is not excluded, $\iota \to -\infty$ and the perturbed electron system will not relax back into the equilibrium. *ii*) In the limit ${\rm{\Gamma }}\to \infty$ (*τ* → 0), the electron system equilibrates immediately into the ground state and, consequently, *ι* = 0. These limiting properties are consistent with the expression *ι* = −*α* · *τ*. Equation (4) also indicates that the time scale is dictated by $\hslash$ and, consequently, on a femto-second time scale. By using the spectral representation of the Green’s function ${\hat{G}}_{{\rm{\Gamma }}}$, we also show the equivalence between the torque-torque correlation model and the Green’s function model proposed in ref. 32 (see Supplementary Material). Please note that in ref. 32 a spin-spin correlation model is considered. Equations (3) and (4) in terms of Green’s function reads

5 $$\begin{array}{rcl}{\alpha }^{\mu \nu } & = & \frac{g}{\pi {M}_{s}}{\rm{Tr}}\,{\int }_{-\infty }^{\infty }{\rm{d}}\varepsilon \eta (\varepsilon )\int \frac{{\rm{d}}{\boldsymbol{k}}}{{\mathrm{(2}\pi )}^{3}}\\  &  & \times [{\hat{T}}^{\mu }\,\Im {\hat{G}}_{{\rm{\Gamma }}}(\varepsilon ,{\boldsymbol{k}}){({\hat{T}}^{\nu })}^{T}\,\Im {\hat{G}}_{{\rm{\Gamma }}}(\varepsilon ,{\boldsymbol{k}})]\end{array}$$

6 $$\begin{array}{rcl}{\iota }^{\mu \nu } & = & \frac{g\hslash }{\pi {M}_{s}}{\rm{Tr}}\,{\int }_{-\infty }^{\infty }{\rm{d}}\varepsilon f(\varepsilon )\int \frac{{\rm{d}}{\boldsymbol{k}}}{{\mathrm{(2}\pi )}^{3}}\\  &  & \times [{\hat{T}}^{\mu }\,\Im {\hat{G}}_{{\rm{\Gamma }}}(\varepsilon ,{\boldsymbol{k}}){({\hat{T}}^{\nu })}^{T}\,\frac{{\partial }^{2}}{\partial {\varepsilon }^{2}}\Re {\hat{G}}_{{\rm{\Gamma }}}(\varepsilon ,{\boldsymbol{k}})\\  &  & +{\hat{T}}^{\mu }\,\frac{{\partial }^{2}}{\partial {\varepsilon }^{2}}\Re {\hat{G}}_{{\rm{\Gamma }}}(\varepsilon ,{\boldsymbol{k}}){({\hat{T}}^{\nu })}^{T}\,\Im {\hat{G}}_{{\rm{\Gamma }}}(\varepsilon ,{\boldsymbol{k}})].\end{array}$$

To obtain materials specific values of the inertia and damping, we performed a Slater-Koster parameterised tight binding (TB) calculations of the torque-torque correlation model as well as for the Green’s function model as described by Eqs (4) and (6), respectively. Here, the TB parameters have been obtained by fitting the electronic structures to those of a first-principles fully relativistic multiple scattering Korringa-Kohn-Rostoker (KKR) method using a genetic algorithm. This puts our model on a firm, first-principles ground. Details of the torque-torque correlation and Green’s function calculations are shown in the Supplemental Material. The materials investigated in this article are bcc Fe, fcc Co, and fcc Ni. Since our magnetic moment is fixed in the *z* direction, variations occur primarily in *x* or *y* directions and, consequently, the effective torque matrix element is ${T}^{-}=\langle n,{\boldsymbol{k}}|[{\sigma }^{-},{H}_{soc}]|m,{\boldsymbol{k}}\rangle$, where *σ* ^−^ = *σ*_*x*_ − i*σ*_*y*_. The cubic symmetry of the selected materials allows only diagonal elements in both damping and moment of inertia tensor.

**Figure 2:**
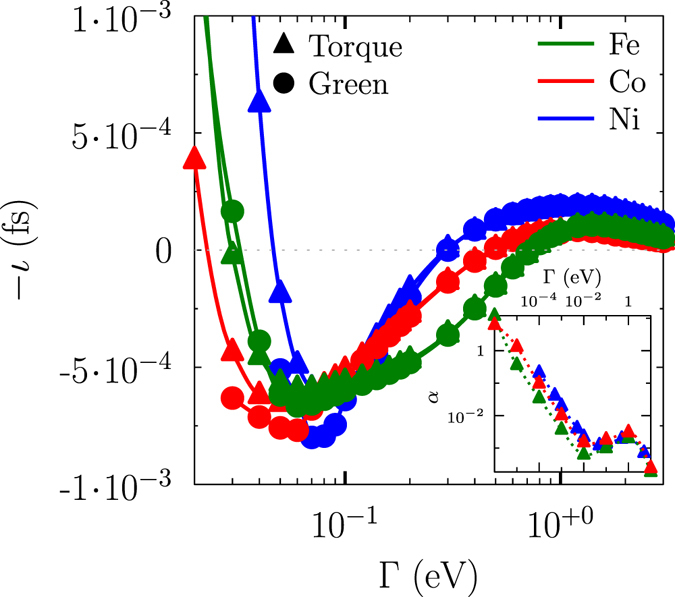
Moment of inertia *ι* as a function of the broadening of the quasiparticle spectral function ${\rm{\Gamma }}$ for bcc Fe (green dotes and lines), fcc Co (red dotes and lines), and fcc Ni (blue dotes and lines) and with two different methods: (*i*) the torque-torque correlation method (filled triangles) and the (*ii*) Greens function method^32^ (filled circles). The dotted grey lines indicating the zero level. The insets show the calculated Gilbert damping *α* as a function of ${\rm{\Gamma }}$. Lines are added to guide the eye. Notice the negative sign of the moment of inertia.

The numerical calculations (Greens function approach and torque-torque correlation model), as shown in Fig. 2, give results that are consistent with the torque-torque correlation model predictions in both limits, ${\rm{\Gamma }}\to 0$ and ${\rm{\Gamma }}\to \infty$. Note that the latter is only true if we assume the validity of the adiabatic limit up to *τ* = 0. It should also be noted that Eq. (4) is only valid in the adiabatic limit (>10 fs). The strong dependency on ${\rm{\Gamma }}$ indicates, however, that the current model is not a parameter-free approach. Fortunately, the relevant parameters can be extracted from *ab*-*initio* methods^42, 43^.

The approximation *ι* = −*α* · *τ* derived by Fähnle *et al*.^35^ from the Kamberský model is not valid for all ${\rm{\Gamma }}$. It holds for ${\rm{\Gamma }}\mathop{ < }\limits_{ \tilde {}}10\,{\rm{meV}}$, where intra-band transitions dominate for both damping and moment of inertia; bands with different energies narrowly overlap. Here, the moment of inertia decreases proportional to $1/{{\rm{\Gamma }}}^{4}$ up to a certain minimum. Above the minimum and with an appropriate large broadening of the quasiparticle spectral function ${\rm{\Gamma }}$, inter-band transitions happen so that the moment of inertia approaches zero for high values of ${\rm{\Gamma }}$. In this range, the relation *ι* = *α* · *τ* used by Ciornei *et al*.^23^ holds, where the sign change in relation to the expression of Fähnle *et al*.^37^ should be noted, and it softens the FMR resonance frequency. Comparing qualitative the difference between the itinerant magnets Fe, Co and Ni, we obtain similar features in *ι* and *α* vs. ${\rm{\Gamma }}$ (see Fig. 2), but the position of the maximum and the slope in the intraband region varies with the elements: *ι*_max_ = 5.9 · 10^−3^ fs at ${\rm{\Gamma }}=60\,{\rm{meV}}$ for bcc Fe, *ι*_max_ = 6.5 · 10^−3^ fs at ${\rm{\Gamma }}=50\,{\rm{meV}}$ for fcc Co, and *ι*_max_ = 6.1 · 10^−3^ fs at ${\rm{\Gamma }}=80\,{\rm{meV}}$ for fcc Ni. The crossing point of intra- to inter-band transitions for the damping was already reported by Gilmore *et al*.^38^ and Thonig *et al*.^44^ — reported also elsewhere^44^ that both studies are in good agreement —, and the results of Fig. 2 show that the inertia has a similar behaviour.

The same trends are also reproduced by applying the Green’s function formalism in ﻿﻿Eqs (5) and (6) (see Fig. 2). Consequently, both methods — torque-torque correlation and the linear response Green’s function method — are equivalent as it can also be demonstrated not only for the moment of inertia but also for the Gilbert damping *α* (see Supplementary Material)^44^. In the torque-torque correlation model (4), the coupling ${\rm{\Gamma }}$ defines the width of the energy window in which transitions *T*_*nm*_ take place. A finite ${\rm{\Gamma }}$ in the Green’s function approach broadens and slightly shifts maxima in the spectral function, which provides a more accurate description with respect to *ab*-*initio* results than the torque-torque correlation approach. In particular, shifted electronic states at energies around the Fermi level causes differences in the minimum of *ι* in both models. Furthermore, the moment of inertia can be resolved by an orbital decomposition and, like the Gilbert damping *α*, scales quadratically with the spin-orbit coupling $\zeta$, caused by the torque operator $\hat{T}$ in Eq. (4). Thus, one criteria for finding large moments of inertia is by having materials with strong spin-orbit coupling.

**Figure 3:**
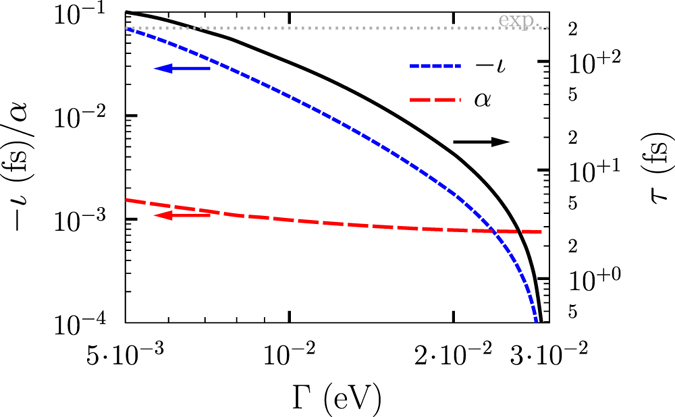
Gilbert damping *α* (red dashed line), moment of inertia *ι* (blue dashed line), and the resulting electron (Bloch-state) lifetime *τ* = −*ι*/*α* (black line) as a function of ${\rm{\Gamma }}$ in the intra-band region for Fe bulk. Arrows indicate the ordinate axis. The grey dotted line shows the experimental value from ref. 31. Notice the negative sign of the moment of inertia.

In order to show the region of ${\rm{\Gamma }}$ where the approximation *ι* = −*α* · *τ* holds, we show in Fig. 3 calculated values of *ι*, *α*, and the resulting Bloch state lifetime *τ* for a selection of ${\rm{\Gamma }}$ that are below *ι*_max_. According to the data reported in ref. 31, this is a suitable regime accessible for experiments. To achieve the room temperature measured experimental values of *τ* = 0.12–0.47 ps, we have furthermore to guarantee that $\iota \gg \alpha$. An appropriate experimental range is ${\rm{\Gamma }}\approx 5-10\,{\rm{meV}}$, which is realistic and caused, e.g., by the electron-phonon coupling. A theoretical value of the Bloch state relaxation time of $\tau \approx 0.25-0.1\,{\rm{ps}}$ is observed for these values of ${\rm{\Gamma }}$ (see Fig. 3), a value similar to that found in experiment. To the best of our knowledge, this is the first time a connection is made between experimentally reported values of the inertia and a calculated electronic structure. Note that the quality of this comparison depends on details of the band-dispersion, which is a direct outcome of our theory, and the value of the broadening of Bloch spectral functions. The latter is in principal possible to evaluate from theory, but more importantly it is accessible from experimental probes such as angular resolved photoelectron spectroscopy and two-photon electron spectroscopy. Although we have not pursued a detailed investigation of the dispersion dependence of this broadening, we note that the value proposed by us here (5–10 meV) is in reasonable agreement with estimates in the literature^45–47^.

**Figure 4:**
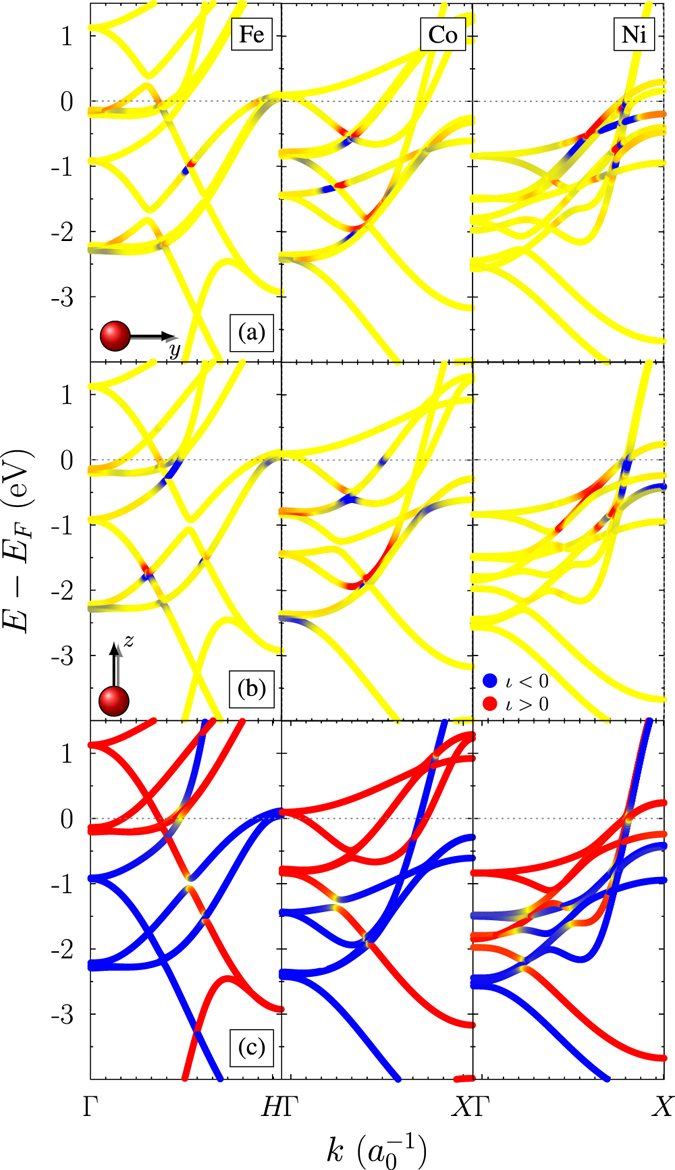
Moment of inertia in the electron band structure for bulk bcc Fe, fcc Co, and fcc Ni with the magnetic moment (**a**) in *y* direction and (**b**) in *z* direction. The colour and the intensity indicates the sign and value of the inertia contribution (blue: *ι* < 0; red: *ι* > 0; yellow: $\iota \approx 0$). The dotted grey line is the Fermi energy and ${\rm{\Gamma }}$ is 0.1 eV. (**c**) Spin polarisation of the electronic band structure (blue - spin down; red - spin up; yellow - mixed states). Note that we analyse for simplicity and clarity only one contribution, *A*_*n*_ *B*_*m*_, in the expression for *V*_*nm*_.

Furthermore, the physical mechanism of magnetic moment of inertia becomes understandable from an inspection of the electron band structure (see Fig. 4). The model proposed here allows to reveal the inertia ***k***- and band-index *n* resolved contributions (integrand of Eq. (4)). Note that we analyse for simplicity and clarity only one contribution, *A*_*n*_ *B*_*m*_, in the expression for *V*_*nm*_. As Fig. 4a,b shows, the contribution to *V*_*nm*_ is significant only for specific energy levels and specific ***k***-points. The figure also shows a considerable anisotropy, in the sense that magnetisations aligned along the z- or y-directions give significantly different contributions in energy and ***k***-space. Also, a closer inspection shows that degenerate or even close energy levels *n* and *m*, which overlap due to the broadening of energy levels, e.g. as caused by electron-phonon coupling, ${\rm{\Gamma }}$, accelerate the relaxation of the electron-hole pairs. This is a consequence of the magnetic moment rotation combined with the spin-orbit coupling. This acceleration decrease the moment of inertia, since inertia is the tendency of staying in a constant magnetic state. Our analysis also shows that the moment of inertia is linked to the spin-polarisation of the bands. Since, as mentioned, the inertia preserves the angular momentum, it has largest contributions in the electronic structure, where multiple electron bands with the same spin-polarisation are close to each other (cf. Fig. 4c). The contribution of the different bands is dictated by the transition matrix elements ${T}_{nm}^{-}$ that are the same for both damping and moment of inertia and obtainable analytically (See Supplementary Material). Here, *p* and *d* states contributing not only by spin flip scattering, but also by spin conservation. Unexpectedly, no transitions within the *e*_*g*_ states as well no transitions from down to up spins are found; the latter is due to *σ* ^−^ in the torque definition. For instance in cobalt, the orbital resolved moment of inertia contribution is caused by the *d* states (contributions from *p* states are three orders of magnitude smaller), where the transitions between *t*_2*g*_ and *e*_*g*_ states contribute with a negative value and transitions between *t*_2*g*_ and *t*_2*g*_ states giving positive contributions. For damping, however, all orbital resolved contributions are positive. The order of magnitude of the absolute value of the orbital resolved contributions are similar for damping and for the moment of inertia.

Some aspects of the inertia, e.g. being caused by band overlaps, are similar to the Gilbert damping^34^. A significant difference is however that the moment of inertia is a property that spans over the whole band structure and not only over the Fermi-surface. Thus, materials with low number of states at the Fermi-level, like FeCo alloys in the appropriate concentration range^48^, as well as half-metallic compounds like Heusler alloys^49^ and chromium dioxide^50^, are likely to have a low damping, but with a significant moment of inertia contribution. Inertia is also relevant in the equation of motion^22, 38^ only for time scales $\mathop{ > }\limits_{ \tilde {}}$0.1 ps and particularly for low dimensional systems. Nevertheless, in the literature there are measurements, as reported in ref. 31, where the inertia effects have also been observed.

**Figure 5:**
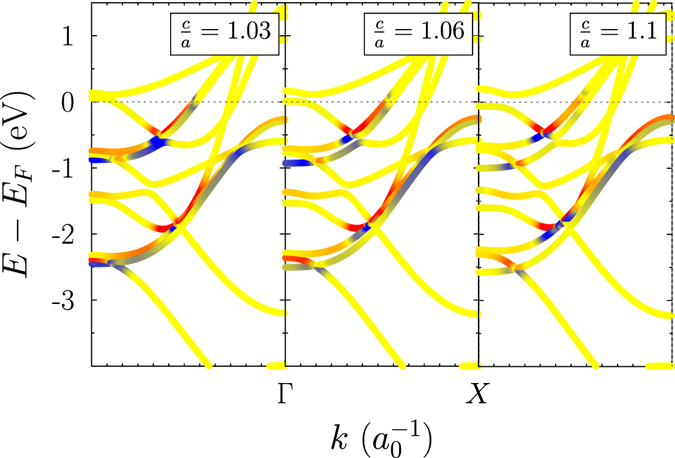
Moment of inertia in the electron band structure for bulk fcc Co strained along the *z* axis. The magnetic moment is also in *z* direction. The colour and the intensity indicates the sign and value of the inertia contribution (blue −*ι* < 0; red −*ι* > 0; yellow −$\iota \approx 0$). The dotted grey line is the Fermi energy and ${\rm{\Gamma }}$ is 0.1 eV. Note that we analyse for simplicity and clarity only one contribution, *A*_*n*_ *B*_*m*_, in the expression for *V*_*nm*_. The integrated moment of inertia is *ι* = 4.78 · 10^−4^ fs, *ι* = 4.88 · 10^−4^ fs, and *ι* = 5.31 · 10^−4^ fs for *c*/*a* = 1.03, *c*/*a* = 1.06, and *c*/*a* = 1.1, respectively.

Understanding the electronic structure origin of the moment of inertia open up the challenge for designing the strength of the moment of inertia, in particular, e.g. the electron band structure can be tuned by straining the crystal structure (Fig. 5), since this lifts degeneracies in the electronic band structure and flattens out particular bands. Another possibility is to influence the width of the spectral function by alloying, where the imaginary part of the self energy coming, e.g., from coherent potential approximation representing ${\rm{\Gamma }}$ ^51^, and this may be the best way to verify the predicted curve in Fig. 2.

The model proposed here, however, has some limitations, since not only the spin-orbit coupling and the underlying electronic structure causes finite moment of inertia. Its derivation from the Kamberský model, Eq. 2, and our numerics corroborates that recently observed properties of the Gilbert damping will be also valid for the moment of inertia. We make the following general observations: *i*) the moment of inertia is temperature dependent^44, 51^ and decays with increasing phonon temperature, where the later usually increase the electron-phonon coupling ${\rm{\Gamma }}$ in certain temperature intervals^45^; *ii*) the moment of inertia is a tensor, however, off-diagonal elements for bulk materials are negligible small; *iii*) it is non-local^39, 44, 52^ and depends on the magnetic moment^53–55^. Note that the sign change of the moment of inertia also effects the dynamics of the magnetic moments (see Supplementary Material). In addition, effects on spin disorder and electron correlation are neglected, that could lead to uncertainties in ${\rm{\Gamma }}$ and hence make the comparison to experiment more difficult. Further, to explain the dynamics of the experiments in ref. 31, it is not excluded that other second order energy dissipation terms, *B*_diss_, proportional to ${(\partial {\boldsymbol{e}}/\partial t)}^{2}$ will also contribute^35^ (see Supplementary Material). This highlights the interesting and rich research field that is coupled to magnetisation inertia, and also the needs for further experimental and theoretical investigations.

In this work, we have derived a theoretical model for the magnetic moment of inertia based on the torque-torque correlation model and found an expression which is similar to that of the Gilbert damping. From numerical calculations we find that the torque-torque correlation model provides identical results to those obtained from a Greens functions formulation. The Gilbert damping and the moment of inertia are both proportional to the spin-orbit coupling, however, the basic electron band structure mechanisms for having inertia are shown to be different than those for the damping. We analyse details of the dispersion of electron energy states, and emphasise the features of a band structure that are important for having a sizeable magnetic inertia. Furthermore, we provide, for the first time, numerical values of the moment of inertia that are comparable with recent experimental measurements^31^. Hence, our total analysis demonstrates analytically and numerically that the *raison d*’*etre* of inertia is to behave opposite to the Gilbert damping. The calculated moment of inertia parameter can be included in atomistic spin-dynamics codes, giving a large step forward in describing ultrafast, sub-ps magnetisation dynamical processes.

## Electronic supplementary material

Magnetic moment of inertia within the torque-torque correlation model —Supplementary Material—
