# Supplementary material for: Magnetic moment of inertia within the torque-torque correlation model
Source: Sci Rep. 2017 Apr 19;7:931. doi: 10.1038/s41598-017-01081-z (PMC5430502; doi:10.1038/s41598-017-01081-z)
Supplement: Supplementary file 1 — Magnetic moment of inertia within the torque-torque correlation model —Supplementary Material— [file 41598_2017_1081_MOESM1_ESM.pdf]

Magnetic moment of inertia within the torque-torque correlation model
—Supplementary Material—

Danny Thonig,* Olle Eriksson, and Manuel Pereiro
Department of Physics and Astronomy, Material Theory, University Uppsala, S-75120 Uppsala, Sweden
(Dated: January 24, 2017)

Contribution from the Division of Materials Theory, Uppsala University. This document is not subject to copyright.

PACS numbers: 75.10.-b,75.30.-m,75.40.Mg,75.78.-n,75.40.Gb

Moment of inertia from Kamberský's breathing Fermi surface model

The breathing Fermi surface model introduced by Kamberský [1, 2] was invented to derive [3] and analyse [4–6] the Gilbert damping tensor from first principles. Thus, it serves as the basis for the derivation of the moment of inertia tensor. Lets consider the non-adiabatic distribution $n_{m\boldsymbol{k}}(t)$ of the electronic state $|m\boldsymbol{k}\rangle$ with energy $\varepsilon_{m\boldsymbol{k}}$ that is perturbed by the non-equilibrium position of the magnetic moments $\boldsymbol{e}$ due to the spin orbit coupling. Here m and $\boldsymbol{k}$ refer to the band index and the reciprocal lattice vector, respectively. The quantum statistical distribution $n_{m\boldsymbol{k}}(t)$ is the Fermi-Dirac distribution $f_{m\boldsymbol{k}}(t) = f(\varepsilon_{m\boldsymbol{k}}(t))$ in equilibrium and its evolution is approached by a relaxation time ansatz [7] with the exact solution

$$n_{m\boldsymbol{k}}(t) = \int_{t_0}^{t} \frac{1}{\tau_{m\boldsymbol{k}}} f_{m\boldsymbol{k}}(t')\, \mathrm{e}^{-\frac{t-t'}{\tau_{m\boldsymbol{k}}}}\, \mathrm{d}t' + n_{m\boldsymbol{k}}(t_0)\, \mathrm{e}^{-\frac{t-t_0}{\tau_{m\boldsymbol{k}}}}. \quad (1)$$

Here, $\tau_{m\boldsymbol{k}}$ is the relaxation time parameter. Using Taylor expansion for $f_{m\boldsymbol{k}}(t')$ around t up to order q, the Kamberský model provides the following expression for the distribution function

$$n_{m\boldsymbol{k}}(t) = \sum_{q=0} (-1)^q\, \tau_{m\boldsymbol{k}}^q \frac{\mathrm{d}^q}{\mathrm{d}t^q} f_{m\boldsymbol{k}}. \quad (2)$$

Gilbert et al. [8] phenomenologically derived a Reighly dissipation field $\boldsymbol{B} = -1/M\, \partial F_{\mathrm{diss}}/\partial \boldsymbol{e}$ in the Lagrangian of the equation of motion for the magnetic moment, taking into account the variation of the free energy F_{diss} by tilting the magnetic moment $\boldsymbol{e}$. Using the magnetic force theorem [9–11],

$$\boldsymbol{B} = -\frac{1}{M} \sum_{m\boldsymbol{k}} n_{j\boldsymbol{k}}(t)\, \frac{\partial \varepsilon_{m\boldsymbol{k}}\left(\boldsymbol{e}\left(t\right)\right)}{\partial \boldsymbol{e}} \quad (3)$$

$$= \boldsymbol{\alpha} \frac{\partial \boldsymbol{e}}{\partial t} - \boldsymbol{\kappa} \frac{\partial \boldsymbol{e}}{\partial t} \frac{\partial \boldsymbol{e}}{\partial t} - \tilde{\kappa}\left(\frac{\partial \boldsymbol{e}}{\partial t}\right) - \boldsymbol{\iota} \frac{\partial^2 \boldsymbol{e}}{\partial t^2} + \ldots, \quad (4)$$

will give a damping tensor $\boldsymbol{\alpha}$ in first order Taylor expansion

$$\alpha^{\mu\nu} = \frac{1}{M} \sum_{m\boldsymbol{k}} \frac{\partial f_{m\boldsymbol{k}}}{\partial \varepsilon_{m\boldsymbol{k}}} \frac{\partial \varepsilon_{m\boldsymbol{k}}}{\partial e^\mu} \frac{\partial \varepsilon_{m\boldsymbol{k}}}{\partial e^\nu} \tau_{m\boldsymbol{k}} \quad (5)$$

and moment of inertia tensor $\boldsymbol{\iota}$ in second order Taylor expansion

$$\iota^{\mu\nu} = -\frac{1}{M} \sum_{m\boldsymbol{k}} \frac{\partial f_{m\boldsymbol{k}}}{\partial \varepsilon_{m\boldsymbol{k}}} \frac{\partial \varepsilon_{m\boldsymbol{k}}}{\partial e^\mu} \frac{\partial \varepsilon_{m\boldsymbol{k}}}{\partial e^\nu} \tau_{m\boldsymbol{k}}^2. \quad (6)$$

as also derived in Ref. [12]. Note that the definition of the damping-like third order tensor $\boldsymbol{\kappa}$ and the functional contribution $\tilde{\kappa}\left(\frac{\partial \boldsymbol{e}}{\partial t}\right)$ are

$$\kappa^{\mu\nu\delta} = -\frac{1}{M}\sum_{m\boldsymbol{k}} \frac{\partial f_{m\boldsymbol{k}}}{\partial \varepsilon_{m\boldsymbol{k}}} \frac{\partial \varepsilon_{m\boldsymbol{k}}}{\partial e^{\mu}} \frac{\partial^2 \varepsilon_{m\boldsymbol{k}}}{\partial e^{\nu}\partial e^{\delta}} \tau^2_{m\boldsymbol{k}}, \tag{7}$$

$$\tilde{\kappa}\left(\frac{\partial \boldsymbol{e}}{\partial t}\right) = -\frac{1}{M}\sum_{m\boldsymbol{k}} \frac{\partial^2 f_{m\boldsymbol{k}}}{\partial \varepsilon^2_{m\boldsymbol{k}}} \left(\frac{\partial \varepsilon_{m\boldsymbol{k}}}{\partial \boldsymbol{e}} \cdot \frac{\partial \boldsymbol{e}}{\partial t}\right)^2 \tau^2_{m\boldsymbol{k}}, \tag{8}$$

in agreement with Ref. 12, but they are not considered in this paper. In our notation $\mu, \nu, \delta \in \{x, y, z\}$. The magnetic force theorem is valid only in the adiabatic limit. Magnetic inertia, however, considers effect in the equation of motion for the magnetic moments that are close to this limit. As an approximation, however, we keep the magnetic force theorem, as assumed also by other authors [13, 14]. Finally, the linear relaxation time approach ($\tau_{m\boldsymbol{k}} \to \tau$) leads to $\boldsymbol{\iota} = -\boldsymbol{\alpha}\tau$. This relation characterizes the nature of inertia as tendency to stay in a certain state and to conserve the total angular momentum.

Magnetic susceptibility and moment of inertia from perturbation theory

Ciornei et al. [14] derived from semi-classical magnetic Boltzmann equation the modified Landau-Lifshitz-Gilbert equation for a macrospin $\boldsymbol{M}$. However, this wok does not comment on how to measure the moment of inertia in experiment. We note, as an example, that via ferromagnetic resonance (FMR) techniques it is possible to get information about the magnetic susceptibility χ out of the Landau-Lifshitz-Gilbert equation

$$\begin{aligned}\frac{\mathrm{d}\boldsymbol{M}}{\mathrm{d}t} &= -\gamma \boldsymbol{M} \times \boldsymbol{B} + \frac{1}{m_s}\boldsymbol{M} \times \left[\boldsymbol{\alpha}\frac{\mathrm{d}\boldsymbol{M}}{\mathrm{d}t}\right] \\ &+ \frac{1}{m_s}\boldsymbol{M} \times \left[\boldsymbol{\iota}\frac{\mathrm{d}^2\boldsymbol{M}}{\mathrm{d}t^2}\right].\end{aligned} \tag{9}$$

where γ is the gyromagnetic ratio. For simplicity, we focus on bulk properties and, consequently, $\boldsymbol{\alpha}$ and $\boldsymbol{\iota}$ as scalar values. When connecting theory to FMR experiments, it is reasonable to linearize the magnetization vector with the approximation of small amplitude dynamics. The magnetization is assumed to point in the z-direction and have a small oscillating transversal response $\boldsymbol{M} = (m_x, m_y, M_s)$, where M_s is the saturation magnetization and $m_{x,y} << M_s$. We set the field $B = (B_x - Nm_x, B_y - Nm_y, B_0 - NM_s)$ in the z-direction with an external-magnetic-field strength B_0 with small transverse oscillating component $B_{x,y}$ in the plane, coming from atomistic interaction (Heisenberg exchange) and in accordance to the experimental setup. The demagnetization tensor $\mathbf{N}$ is regarded to account for dipole-dipole interaction between the macrospins $\boldsymbol{M}$. Lets consider that the precession frequency ω of the magnetic moment $\boldsymbol{M}$ is due to the field $\boldsymbol{B}$. We obtain an expression for the magnetic susceptibility, defined by $\boldsymbol{M} = \boldsymbol{\chi}\boldsymbol{B}$, as

$$\begin{aligned}\boldsymbol{\chi} = &\frac{1}{(\gamma B_0 - \mathrm{i}\alpha\omega - \iota\omega^2)^2 - \omega^2} \times \\ &\begin{pmatrix} \gamma M_s(\gamma B_0 - \mathrm{i}\alpha\omega - \iota\omega^2) & -\mathrm{i}\omega\gamma M_s \\ \mathrm{i}\omega\gamma M_s & \gamma M_s(\gamma B_0 - \mathrm{i}\alpha\omega - \iota\omega^2) \end{pmatrix},\end{aligned} \tag{10}$$

where in experiment typically the transverse susceptibility $\chi^{\perp}$ (diagonal elements of the matrix $\boldsymbol{\chi}$) is probed. Here, $\omega_m = \gamma M_s$, $\omega_0 = \gamma B_0$ and α, ι are considered only in first order, since $\alpha, \iota << 1$. To extract the Gilbert damping parameter, we consider the imaginary part of $\chi^{\perp}$ in the limit when ω goes to zero [15]. The moment of inertia ι does not affect the imaginary part of the response function, however, it is defined by the first derivative of the real part of the response function, and we obtain the following expressions for inertia and damping parameters:

$$\alpha = \frac{\omega_0^2}{\omega_M} \lim_{\omega\to 0} \frac{\Im\chi^{\perp}}{\omega} \tag{11}$$

$$\iota = \frac{1}{2}\frac{\omega_0^2}{\omega_M} \lim_{\omega\to 0} \frac{\partial_\omega \Re\chi^{\perp}}{\omega} - \frac{1}{\omega_0}. \tag{12}$$

This result does not conserve the sign convention $\iota = -\alpha\tau$ predicted by the Kamberský model [12], something which is due to the sense of precession that we assume in the derivative ${}^{\partial \boldsymbol{m}}/_{\partial t}$. The opposite rotation, however, will conserve Kamberský 's findings.

Experimentally, however, it is more reasonable to consider rapid changes of the external [16] or spin-current induced field [17] B_0. Here, the moment of inertia is $\iota = -\frac{1}{6}\frac{\omega_0^2}{\omega_M}\left[\left(\lim_{\omega\to 0}\frac{\Re\chi^{\perp}(B_0)}{\omega^2}\right) + \left(\lim_{\omega\to 0}\frac{\Re\chi^{\perp}(-B_0)}{\omega^2}\right)\right]$. Note, that inertia is not measurable by triggering the probe field ω, since ω enters quadratic into the real part of the response function.

Torque-torque-correlation model

Our ultimate goal is to calculate the response function in Eq. (12) and, consequently, the inertia from first principles. The magnetic susceptibility is accessible via the Kubo-Greenwood formalism [18] for the Green's function $\mathcal{G}$ and define as a spin-spin correlator between two Pauli matrices σ^{μ}:

$$\begin{aligned}\chi\left(\boldsymbol{q},\omega\right) &= -\hbar\gamma^2\mathcal{G}\left(\sigma^{\dagger},\sigma,\omega\right)\\ &= \mathrm{i}\hbar\gamma^2\int_{-\infty}^{\infty}\mathrm{e}^{-\mathrm{i}\omega\tau}\Theta(\tau)\left\langle\left\langle\sigma^{\dagger}(\boldsymbol{q},\tau),\sigma(\boldsymbol{q},0)\right\rangle\right\rangle\mathrm{d}\tau.\end{aligned} \tag{13}$$

Here, γ is the gyromagnetic ratio. Since we want to derive the change of the eigenstates with respect to small tilting, the spin is not a good quantity, since it has a fixed length. Thus, it is more reasonable to consider the variation in terms of torques $T^{\mu}(\tau) = [\sigma^{\mu}(\tau), H]$. The electron hamiltonian H splits in a spin-polarized part H_{sp} and a spin-orbit part H_{soc}, where damping and moment of inertia are related only to the latter. The spin polarized part causes only precession $[\sigma^{\mu}, H_{sp}] = \hbar\Omega\sigma^{\mu}$ with frequency Ω, where $\mu = x, y$. Gilmore [15] showed in detail that the response function $\chi^{\perp}(\boldsymbol{q},\omega)$ in first order expansion of the Dyson equation (random phase approximation; RPA [19]) reads

$$\chi^{\perp}\left(\boldsymbol{q},\omega\right) = -\hbar\omega_M\frac{\mathcal{G}(\sigma^{-},\sigma^{+},\omega)}{\hbar + \Sigma\mathcal{G}(\sigma^{-},\sigma^{+},\omega)}, \tag{14}$$

an expression which makes use of the mean field exchange frequency Δ, $\Sigma = {}^{\hbar\Delta}/_{2\langle\sigma^z\rangle}$, and the Green's function for the spin-spin correlation

$$\mathcal{G}(\sigma^{-},\sigma^{+},\omega) = \frac{\Delta\Sigma^{-1}}{(\omega-\Omega)} + \frac{\mathcal{G}(T^{-},T^{+},\omega)}{\hbar^2(\omega-\Omega)^2}. \tag{15}$$

In the following, we set $\mathcal{G} = \mathcal{G}(T^{-},T^{+},\omega)$ and $\Omega = 0$. To satisfy Eq. (12), the real part $\Re\chi^{\perp}$ and the imaginary part $\Im\chi^{\perp}$ of the response function must be separated. The spin-orbit torque vanishes as the spin motion ceases; thus, the torque response function $\mathcal{G}$ goes to zero in the limit that the frequency goes to zero. However, this is not necessarily valid for the derivative of $\mathcal{G}$. We collect terms that are constant and of first order in $\partial_{\omega}\Re\mathcal{G}$ and let $\omega\to 0$

$$\lim_{\omega\to 0}\partial_{\omega}\Re\chi^{\perp} = -\frac{\omega_M}{\hbar\omega_0^2}\lim_{\omega\to 0}\frac{\partial_{\omega}\Re\mathcal{G}}{\omega} + \frac{\omega_M\Delta\Sigma^{-1}}{\omega_0^2}, \tag{16}$$

where $\omega_0 = \Delta - \Omega$. Gilmore [15] showed that the torque-torque correlation function $\mathcal{G}$ is

$$\begin{aligned}\mathcal{G}\left(\boldsymbol{q},\omega\right) = \sum_{nm}\int\frac{\mathrm{d}\boldsymbol{k}}{(2\pi)^3}\int\frac{\mathrm{d}\boldsymbol{k}'}{(2\pi)^3}\left|T^{-}_{nm}\left(\boldsymbol{k},\boldsymbol{q}\right)\right|^2\\ \int_{-\infty}^{\infty}\mathrm{d}\varepsilon\int_{-\infty}^{\infty}\mathrm{d}\varepsilon'\left[f\left(\varepsilon\right)-f\left(\varepsilon'\right)\right]A_{n\boldsymbol{k}}\left(\varepsilon\right)A_{m\boldsymbol{k}'}\left(\varepsilon'\right)\\ \frac{1}{(\hbar\omega+\varepsilon-\varepsilon')+\mathrm{i}\delta},\end{aligned} \tag{17}$$

where $\left|\langle n\boldsymbol{k}|T^{-}\left(\boldsymbol{q}\right)|m\boldsymbol{k}'\rangle\right|^2 = \left|T^{-}_{nm}\left(\boldsymbol{k},\boldsymbol{q}\right)\right|^2$, $f(\varepsilon)$ is the Fermi-Dirac distribution function and the Lorentzian spectral function $A_{n\boldsymbol{k}}$ approaches a δ-function. The first derivative of the correlator (17) with respect to ω acts only on the last

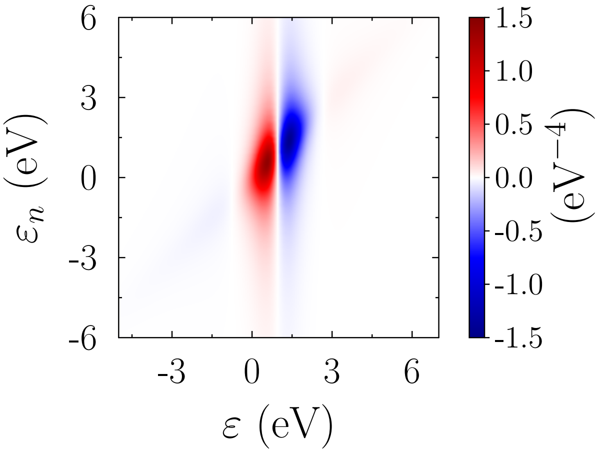

FIG. 1: (Color online) The weighting factor $\Im K_{n\boldsymbol{k}}\left(\varepsilon\right)\partial^2_{\varepsilon^2}\Re K_{m\boldsymbol{k}}(\varepsilon)$ as a function of energy ε and $\varepsilon_{n\boldsymbol{k}} - \varepsilon_{m\boldsymbol{k}}$, where $\varepsilon_{m\boldsymbol{k}} = 1\,\mathrm{eV}$ and $\Gamma = 1\,\mathrm{eV}$. Note that the plot is made in form of an energy landscape where red indicated large positive contributions and blue large negative contributions.

term. For the energy integrations we define $K_{m\boldsymbol{k}}$, where $\Im K_{m\boldsymbol{k}} = -\pi A_{m\boldsymbol{k}}$ and apply the Sokhotski-Plemelj theorem [20]

$$\lim_{\omega\to 0}\frac{1}{\omega}\partial_\omega \Re\mathcal{G}\left(\omega\right) = \frac{1}{\pi}\sum_{nm}\int\frac{\mathrm{d}\boldsymbol{k}}{(2\pi)^3}\left|T^-_{nm}\left(\boldsymbol{k}\right)\right|^2 \int_{-\infty}^{\infty}\mathrm{d}\varepsilon f\left(\varepsilon\right)\left[\Im K_{n\boldsymbol{k}}\left(\varepsilon\right)\frac{\partial^2}{\partial\varepsilon^2}\Re K_{m\boldsymbol{k}}\left(\varepsilon\right) + \Im K_{m\boldsymbol{k}}\left(\varepsilon\right)\frac{\partial^2}{\partial\varepsilon^2}\Re K_{n\boldsymbol{k}}\left(\varepsilon\right)\right]. \tag{18}$$

For simplification we consider just $\boldsymbol{k'} = \boldsymbol{k}$, as Gilmore did for the expression of damping [15]. For $K_{m\boldsymbol{k'}}$, we get

$$\Re K_{m\boldsymbol{k}}\left(\varepsilon\right) = \frac{\varepsilon - \varepsilon_{m\boldsymbol{k}}}{\left(\varepsilon - \varepsilon_{m\boldsymbol{k}}\right)^2 + \Gamma^2} \tag{19}$$

$$\Im K_{m\boldsymbol{k}}\left(\varepsilon\right) = -\frac{\Gamma}{\left(\varepsilon - \varepsilon_{m\boldsymbol{k}}\right)^2 + \Gamma^2} \tag{20}$$

and, consequently, the second derivative with respect to ε is

$$\partial^2_{\varepsilon^2}\Re K_{m\boldsymbol{k}}(\varepsilon) = \frac{2(\varepsilon - \varepsilon_{m\boldsymbol{k}})\left((\varepsilon - \varepsilon_{m\boldsymbol{k}})^2 - 3\Gamma^2\right)}{\left(\left(\varepsilon - \varepsilon_{m\boldsymbol{k}}\right)^2 + \Gamma^2\right)^3} \tag{21}$$

Figure 1 shows $\Im K_{n\boldsymbol{k}}\left(\varepsilon\right)\partial^2_{\varepsilon^2}\Re K_{m\boldsymbol{k}}(\varepsilon)$, i.e. part of the integrand in Eq. 18, revealing a complex behavior of the broadening of the bands, where for certain energies even a negative contribution may be seen.

In summary, the inertia reads

$$\iota = \frac{g\hbar}{M_s\pi}\sum_{nm}\int\frac{\mathrm{d}\boldsymbol{k}}{(2\pi)^3}\left|T^-_{nm}\left(\boldsymbol{k}\right)\right|^2 \int_{-\infty}^{\infty}\mathrm{d}\varepsilon f\left(\varepsilon\right)\left[\Im K_{n\boldsymbol{k}}\left(\varepsilon\right)\frac{\partial^2}{\partial\varepsilon^2}\Re K_{m\boldsymbol{k}}\left(\varepsilon\right) + \Im K_{m\boldsymbol{k}}\left(\varepsilon\right)\frac{\partial^2}{\partial\varepsilon^2}\Re K_{n\boldsymbol{k}}\left(\varepsilon\right)\right]. \tag{22}$$

Note that the inertia tensor $\iota^{\mu\nu}$ can be introduced by replacing T^- by T^μ, where $\mu \in \{x, y, z\}$.

	$\lvert p_x,\uparrow\rangle$	$\lvert p_y,\uparrow\rangle$	$\lvert p_z,\uparrow\rangle$	$\lvert p_x,\downarrow\rangle$	$\lvert p_y,\downarrow\rangle$	$\lvert p_z,\downarrow\rangle$
$\langle p_x,\uparrow\rvert$	0	0	$-\hbar^2\xi$	0	0	0
$\langle p_y,\uparrow\rvert$	0	0	$\mathrm{i}\hbar^2\xi$	0	0	0
$\langle p_z,\uparrow\rvert$	$\hbar^2\xi$	$\mathrm{i}\hbar^2\xi$	0	0	0	0
$\langle p_x,\downarrow\rvert$	0	$\frac{\mathrm{i}\hbar^2\xi}{2}$	0	0	0	$\hbar^2\xi$
$\langle p_y,\downarrow\rvert$	$\frac{\mathrm{i}\hbar^2\xi}{2}$	0	0	0	0	$-\mathrm{i}\hbar^2\xi$
$\langle p_z,\downarrow\rvert$	0	0	0	$-\hbar^2\xi$	$-\mathrm{i}\hbar^2\xi$	0

TABLE I: Transition matrix elements T^-_{nm} for p states.

Equivalence between Torque-torque-correlation model and Green's function method

As showed by Thonig et al. [6] and Sakuma et al. [21], the torque-torque correlation model from Gilmore et al. [3] and the linear response model from Ebert et al. [22] are equivalent. The same is for the here derived torque-torque correlation model and the linear response Green's function formalism of Bhattacharjee et al. [13]. For this purpose we rewrite $|T^-_{nm}|^2 = \langle n\boldsymbol{k}|\hat{T}^-|m\boldsymbol{k}\rangle\langle m\boldsymbol{k}|(\hat{T}^-)^T|n\boldsymbol{k}\rangle$. Using the Lehman representation of the electron Green's function $\hat{G}_\Gamma(\varepsilon,\boldsymbol{k}) = \sum_n \frac{|n\boldsymbol{k}\rangle\langle n\boldsymbol{k}|}{(\varepsilon-\varepsilon_{n\boldsymbol{k}}+\mathrm{i}\Gamma)}$, the moment of inertia reads

$$\begin{aligned}\iota = \frac{g\hbar}{\pi M_s}\mathrm{Tr}\int_{-\infty}^{\infty}\mathrm{d}\varepsilon f(\varepsilon)\int\frac{\mathrm{d}\boldsymbol{k}}{(2\pi)^3} \\ \Bigg[\hat{T}^-\,\Im\hat{G}_\Gamma(\varepsilon,\boldsymbol{k})\,(\hat{T}^-)^T\frac{\partial^2}{\partial\varepsilon^2}\Re\hat{G}_\Gamma(\varepsilon,\boldsymbol{k}) + \\ \hat{T}^-\frac{\partial^2}{\partial\varepsilon^2}\Re\hat{G}_\Gamma(\varepsilon,\boldsymbol{k})\,(\hat{T}^-)^T\,\Im\hat{G}_\Gamma(\varepsilon,\boldsymbol{k})\Bigg].\end{aligned} \tag{23}$$

Note that the second derivative of the real part of the electron Greens function is $\partial^2_{\varepsilon^2}\Re\hat{G} = 2\left((\Re\hat{G})^3 - \Re\hat{G}(\Im\hat{G})^2 - \Im\hat{G}\Re\hat{G}\Im\hat{G} - (\Im\hat{G})^2\Re\hat{G}\right)$.

Transition element matrix

With the focus on the electronic band structure as the origin for high moment of inertia, it is vital to study possible allowed transitions between the bands, say by evaluating the transition matrix elements $T^-_{nm} = \langle n|\,[\sigma^-, H_{soc}]\,|m\rangle$. Here, we express $\hat{T}$ in terms of ladder operators $S^\pm, L^\pm$ and S^z, L^z, reads

$$\hat{T} = \frac{\xi}{2\hbar}\left(2\hat{S}^-\hat{L}^-\hat{S}^+ + \hat{S}^-\hat{L}^z\hat{S}^z - 2\hat{L}^-\hat{S}^+\hat{S}^- - \hat{L}^z\hat{S}^z\hat{S}^-\right) \tag{24}$$

and apply it to to basis functions in spherical harmonics that are finally transformed into cubic harmonics for p states (Table I) and d-states (Table II).

Both p and d bands will contribute to the damping and the moment of inertia either by spin-flip scattering or by spin conservations.

Tight Binding method

The tight binding (TB) method used in the current article is based on the Slater-Koster parametrization [23, 24] in a linear combination of atomic sp^{3}d^5-orbitals (LCAO). The TB parameters are obtained by fitting the TB band structures to ab initio band structures coming from a fully relativistic multiple-scattering Green's function approach (KKR method) [25] (see Table III). For the fitting, we apply a genetic algorithm [26] and Monte Carlo fitting [27] with an accuracy better than 10^{-4} eV. The parameters are in good agreement with those reported in Refs. 28–31.

The tight binding Hamiltonian $\mathcal{H} = \mathcal{H}_0 + \mathcal{H}_{mag} + \mathcal{H}_{soc}$ contains on-site energies and hopping elements $\mathcal{H}_0$, the spin-orbit coupling $\mathcal{H}_{soc} = \zeta\boldsymbol{S}\cdot\boldsymbol{L}$ [32] and the Zeeman term $\mathcal{H}_{mag} = {}^1\!/\!_2\boldsymbol{B}\cdot\boldsymbol{\sigma}$. The Green's function is obtained by

	$\|d_{xy},\uparrow\rangle$	$\|d_{yz},\uparrow\rangle$	$\|d_{zx},\uparrow\rangle$	$\|d_{x^2-y^2},\uparrow\rangle$	$\|d_{z^2-r^2},\uparrow\rangle$	$\|d_{xy},\downarrow\rangle$	$\|d_{yz},\downarrow\rangle$	$\|d_{zx},\downarrow\rangle$	$\|d_{x^2-y^2},\downarrow\rangle$	$\|d_{z^2-r^2},\downarrow\rangle$
$\langle d_{xy},\uparrow\|$	0	$\hbar^2\xi$	$\mathrm{i}\hbar^2\xi$	0	0	0	0	0	0	0
$\langle d_{yz},\uparrow\|$	$-\hbar^2\xi$	0	0	$\mathrm{i}\hbar^2\xi$	$\sqrt{3}\mathrm{i}\hbar^2\xi$	0	0	0	0	0
$\langle d_{zx},\uparrow\|$	$\mathrm{i}\hbar^2\xi$	0	0	$\hbar^2\xi$	$-\sqrt{3}\hbar^2\xi$	0	0	0	0	0
$\langle d_{x^2-y^2},\uparrow\|$	0	$\mathrm{i}\hbar^2\xi$	$-\hbar^2\xi$	0	0	0	0	0	0	0
$\langle d_{z^2-r^2},\uparrow\|$	0	$\sqrt{3}\mathrm{i}\hbar^2\xi$	$\sqrt{3}\hbar^2\xi$	0	0	0	0	0	0	0
$\langle d_{xy},\downarrow\|$	0	0	0	$-2\frac{\mathrm{i}\hbar^2\xi}{2}$	0	0	$-\hbar^2\xi$	$-\mathrm{i}\hbar^2\xi$	0	0
$\langle d_{yz},\downarrow\|$	0	0	$-\frac{\mathrm{i}\hbar^2\xi}{2}$	0	0	$\hbar^2\xi$	0	0	$-\mathrm{i}\hbar^2\xi$	$-\sqrt{3}\mathrm{i}\hbar^2\xi$
$\langle d_{zx},\downarrow\|$	0	$\frac{\mathrm{i}\hbar^2\xi}{2}$	0	0	0	$-\mathrm{i}\hbar^2\xi$	0	0	$-\hbar^2\xi$	$\sqrt{3}\hbar^2\xi$
$\langle d_{x^2-y^2},\downarrow\|$	$2\frac{\mathrm{i}\hbar^2\xi}{2}$	0	0	0	0	0	$-\mathrm{i}\hbar^2\xi$	$\hbar^2\xi$	0	0
$\langle d_{z^2-r^2},\downarrow\|$	0	0	0	0	0	0	$-\sqrt{3}\mathrm{i}\hbar^2\xi$	$-\sqrt{3}\hbar^2\xi$	0	0

TABLE II: Transition matrix elements T^{-}_{nm} for d states.

	Fe	Co	Ni
ε_s	6.006	5.322	2.191
ε_p	12.658	14.000	7.311
$\varepsilon_{t_{2g}}$	-0.853	-1.389	-2.495
ε_{e_g}	-0.955	-1.402	-2.634
B_s	0.436	0.329	0.015
B_p	0.793	1.237	0.059
$B_{t_{2g}}$	2.069	1.572	0.636
B_{e_g}	2.034	1.528	0.688
λ_p	0.200	0.100	0.328
λ_d	0.080	0.070	0.133
$(ss\sigma)_1$	-1.494	-1.144	-1.010
$(sp\sigma)_1$	-2.035	-1.708	1.502
$(sd\sigma)_1$	0.769	0.435	-0.839
$(pp\sigma)_1$	2.901	3.113	1.866
$(pp\pi)_1$	-0.112	-0.204	-0.217
$(pd\sigma)_1$	-0.903	-0.233	-1.107
$(pd\pi)_1$	0.303	0.510	0.158
$(dd\sigma)_1$	-0.632	-0.515	-0.495
$(dd\pi)_1$	0.4121	0.387	0.232
$(dd\delta)_1$	-0.066	-0.0927	-0.004
$(ss\sigma)_2$	-0.352	-0.042	-0.082
$(sp\sigma)_2$	-0.924	0.017	0.076
$(sd\sigma)_2$	0.448	0.023	0.016
$(pp\sigma)_2$	2.347	0.041	0.231
$(pp\pi)_2$	-0.145	0.083	0.125
$(pd\sigma)_2$	-0.661	0.138	-0.114
$(pd\pi)_2$	0.125	0.305	-0.062
$(dd\sigma)_2$	-0.384	-0.111	-0.132
$(dd\pi)_2$	0.152	0.082	-0.069
$(dd\delta)_2$	-0.005	0.013	0.023

TABLE III: Tight binding parameters for bulk bcc Fe, fcc Co, (from Ref. 6) and fcc Ni. The notation follows that of Slater and Koster [23] for the on-site energies ε and hopping parameters $(\mu\nu\kappa)_n$ between orbitals μ and ν of bond κ for the nth interaction shell. λ and B stand for orbital dependent spin-orbit coupling strength and exchange splitting, respectively. All values are in eV. Note that only nearest and next-nearest interactions are considered in this calculation.

$\mathcal{G} = (\varepsilon + \mathrm{i}\Gamma - \mathcal{H})^{-1}$, allows to consider disorder in terms of spin and phonon as well as alloys [6]. Despite the fact that the tight binding approach is limited in accuracy, it produces good agreement with first principle band structure calculations for energies $< \varepsilon_F + 5\,\mathrm{eV}$.

Numerical aspects: The moment of inertia calculations are performed for $\boldsymbol{k}$ integration in the first Brillouin zone up to 10^7 (400x400x400) mesh points. The convergence was checked by applying 600x600x600 mesh point. The energy integration from the bottom of the valence band to the Fermi energy ε_F is approximated by about 6400 energy points in Gauss-Legendre quadrature procedure. Electron-lattice interactions were treated phenomenologically by Γ as a broadening of the spectral functions. The number of energy points depends on the parameter Γ; for large $\Gamma > 0.1$

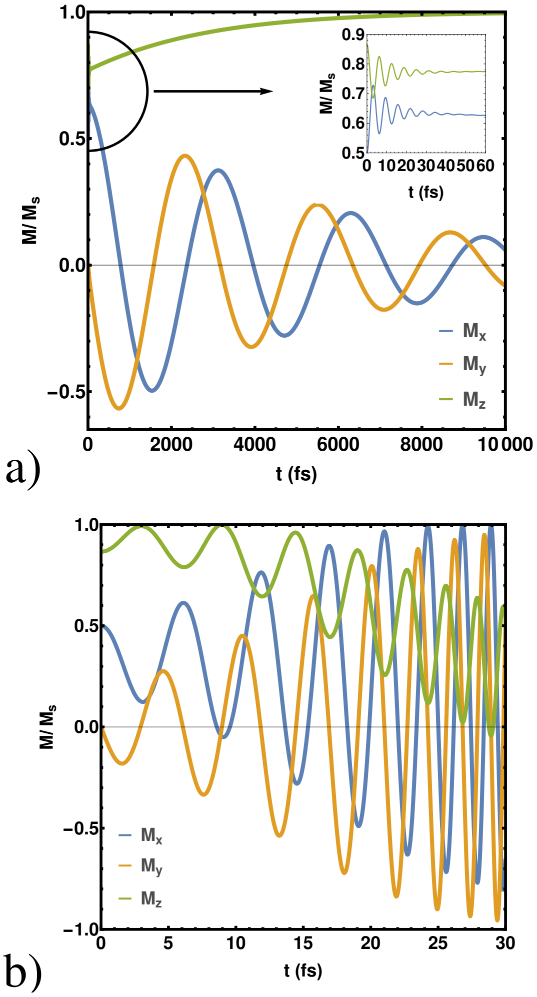

FIG. 2: (Color online) Dynamics of a macrospin $\boldsymbol{M}$ in an external magnetic field with a) negative moment of inertia and b) positive moment of inertia. Different colors indicating different Cartesian components of the magnetization vector (blue - M_x, orange - M_y, green - M_z). The inset shows a zoom to shorter times. Initial conditions are $\theta(0) = {}^{\pi}/_{6}$, $\phi(0) = 0$, $\dot{\theta}(0) = 0$, and $\dot{\phi}(0) = 0.2\sqrt{3}\,\mathrm{fs}^{-1}$.

only 320 points are sufficient. Note that a high number of energy points are required as soon as energy eigenvalues approaches the singularities of the Green's function.

Inertia in magnetization dynamics

The change of the sign in the moment of inertia affects the dynamics of the magnetic moments. To address this issue we solve the extended Landau-Lifshitz-Gilbert equation for a single macrospin within an external magnetic field B along z direction. Here, we use the spherical coordinate representation as shown in Ref. 33 and use $B = 2\,\mathrm{T}$, $\alpha = 0.1$, and $\iota = \pm 1\,\mathrm{fs}$ (Fig. 2).

Note that our definition of the moment of inertia accounts for the opposite rotation sense than in Ref. 33. Thus, our negative moment of inertia would be defined as being positive in Ref. 33. In agreement with previous studies [33, 34] we observe a damped nutation of the magnetic moment up to 100 fs for negative ι. Elongated cycloid was observed rather than abbreviated. For positive inertia, however, the evolution of the moment gets chaotic. This reveals an unphysical limit where the moment "overshoots" into a new magnetic state and will not equilibrate. However, in the region where $\iota > 0$ the damping term is the dominating contribution and it stabilize the dynamics.

* Electronic address: danny.thonig@physics.uu.se

1. Kamberský, V. On the Landau-Lifshitz relaxation in ferromagnetic metals. *Can. J. Phys.* **48**, 2906–2911 (1970).
2. Kamberský, V. FMR linewidth and disorder in metals. *Czech. J. Phys. B* **34**, 1111–1124 (1984).
3. Gilmore, K., Idzerda, Y. U. & Stiles, M. D. Identification of the dominant precession-damping mechanism in Fe, Co, and Ni by first-principles calculations. *Phys. Rev. Lett.* **99**, 027204 (2007).
4. Steiauf, D. & Fähnle, M. Damping of spin dynamics in nanostructures: An *ab initio* study. *Phys. Rev. B* **72**, 064450 (2005).
5. Fähnle, M. & Steiauf, D. Breathing Fermi surface model for noncollinear magnetization: A generalization of the Gilbert equation. *Phys. Rev. B* **73**, 184427 (2006).
6. Thonig, D. & Henk, J. Gilbert damping tensor within the breathing fermi surface model: anisotropy and non-locality. *New J. Phys.* **16**, 013032 (2014).
7. Ashcroft, N. W. & Mermin, N. D. *Solid State Physics* (Holt-Saunders International Editions, London, 1976).
8. Gilbert, T. L. A phenomenological theory of damping in ferromagnetic materials. *IEEE Transact. Magn.* **40**, 6 (2004).
9. Andersen, O. K., Skriver, H. L., Nohl, N. & Johansson, B. Electronic structure of transition metal compounds; ground-state properties of the 3d-monoxides in the atomic sphere approximation. *Pure Appl. Chem.* **52**, 93–118 (1979).
10. Liechtenstein, A. I., Katsnelson, M. I., Antropov, V. P. & Gubanov, V. A. Local spin density functional approach to the theory of exchange interactions in ferromagnetic metals and alloys. *J. Magn. Magn. Mater.* **67**, 65–74 (1987).
11. Bruno, P. Exchange interaction parameters and adiabatic spin-wave spectra of ferromagnets: A "renormalized magnetic force theorem". *Phys. Rev. Lett.* **90**, 8 (2003).
12. Fähnle, M., Steiauf, D. & Illg, C. Erratum: Generalized gilbert equation including inertial damping: Derivation from an extended breathing fermi surface model [phys. rev. b 84, 172403 (2011)]. *Phys. Rev. B* **88**, 219905 (2013).
13. Bhattacharjee, S., Nordström, L. & Fransson, J. Atomistic spin dynamic method with both damping and moment of inertia effects included from first principles. *Phys. Rev. Lett.* **108**, 057204 (2012).
14. Ciornei, M.-C. *Role of magnetic inertia in damped macrospin dynamics.* Ph.D. thesis, Ecole Polytechnique, Universidad de Barcelona (2010).
15. Gilmore, K. *Precession damping in itinerant ferromagnets.* Ph.D. thesis, MONTANA STATE UNIVERSITY (2007).
16. Li, Y., Barra, A. L., Auffret, S., Ebels, U. & Bailey, W. E. Inertial terms to magnetization dynamics in ferromagnetic thin films. *Physical Review B* **92**, 140413 (2015).
17. Zhou, Y. *et al.* Oscillatory transient regime in the forced dynamics of a nonlinear auto oscillator. *Phys. Rev. B* **82**, 012408 (2010).
18. Mahan, G. D. *Many particle physics* (Springer-Verlag, 1981).
19. Mattuck, R. D. *A Guide to Feynman Diagrams in the Many-Body Problem* (Dover, New York, 1976), 2 edn.
20. Plemelj, J. *Problems in the sense of Riemann and Klein* (Interscience Publishers, 1964).
21. Sakuma, A. Theoretical investigation on the relationship between the torque correlation and spin correlation models for the gilbert damping constant. *Journal of Applied Physics* **117**, 013912 (2015).
22. Ebert, H., Mankovsky, S., Ködderitzsch, D. & Kelly, J. P. Ab-initio calculation of the Gilbert damping parameter via linear response formalism. *Phys. Rev. Lett.* **107**, 066603 (2011).
23. Slater, J. C. & Koster, G. F. Simplified LCAO method for the periodic potential problem. *Phys. Rev.* **94**, 1498–1524 (1954).
24. Harrison, W. *Electronic Structure and the Properties of Solids* (W. H. Freeman, San Francisco, 1980).
25. Zabloudil, J., Hammerling, R., Szunyogh, L. & Weinberger, P. (eds.) *Electron Scattering in Solid Matter* (Springer, Berlin, 2005).
26. Goldberg, D. E. (ed.) *Genetic Algorithms in Search, Optimization, and Machine Learning* (Addison-Wesley, 1989).
27. Binder, K. & Heermann, D. W. *Monte Carlo Simulation in Statistical Physics: An Introduction* (Springer, Berlin, 1997), 3 edn.
28. Zhong, W., Overney, G. & Tománek, D. Structural properties of Fe crystal. *Phys. Rev. B* **47**, 95–99 (1993).
29. Liu, G., Nguyen-Manh, D., Liu, B. G. & Pettifor, D. G. Magnetic properities of point defects in iron within the tight-binding-bond stoner model. *Phys. Rev. B* **71**, 174115 (2005).
30. Okutani, M. & Jo, T. Orbital magnetic moment in superlattices of transition metals. *J. Phys. Soc. Jpn.* **69**, 598–606 (2000).
31. Sluiter, M. H. F. & Singh, P. P. Transferable tight-binding parameters: An application to Ni and Ni-Al alloys. *Phys. Rev. B* **49**, 10918–10925 (1994).
32. Schena, T. *Tight-Binding Treatment of Complex Magnetic Structures in Low-Dimensional Systems.* Master's thesis, Rheinisch-Westfälische Technische Hochschule Aachen (2010).
33. Olive, E., Lansac, Y. & Wegrowe, J. E. Beyond ferromagnetic resonance: The inertial regime of the magnetization. *Appl. Phys. Lett.* **100**, 192407 (2012).
34. Böttcher, D. & Henk, J. Significance of nutation in magnetization dynamics of nanostructures. *Phys. Rev. B* **86**, 020404(R) (2012).
